# Behavioral Responses of *Bemisia tabaci* Mediterranean Cryptic Species to Three Host Plants and Their Volatiles

**DOI:** 10.3390/insects13080703

**Published:** 2022-08-05

**Authors:** Zhe Liu, Wenbin Chen, Shuai Zhang, Han Chen, Honghua Su, Tianxing Jing, Yizhong Yang

**Affiliations:** 1 College of Horticulture and Plant Protection, Yangzhou University, Yangzhou 225009, China; 2 Jiangsu Coastal Area Institute of Agricultural Sciences, Yancheng 224002, China

**Keywords:** tobacco whitefly, Mediterranean (MED) cryptic species, selectivity, plant growth stage, volatile compounds, push–pull strategy

## Abstract

**Simple Summary:**

*Bemisia tabaci* Gennadius (Hemiptera: Aleyrodidae) was first observed in tobacco in Greece in 1889. In China, since the introduction of *Euphorbia pulcherrima* (Euphorbiales: Euphorbiaceae, native to Central America) at the end of the previous century, *B. tabaci* has gradually become an increasingly significant agricultural pest. In recent years, the push–pull strategy has been widely applied to the control of the pest. The distinct volatiles are emitted of plants directly affect the efficiency of pushing and pulling; hence, we aimed to study the behavioral responses of *B. tabaci* to three plants (*Gossypium hirsutum, Abutilon theophrasti*, and *Ricinus communis*) during various growth phases (pre-flowering, fluorescence, and fruiting) and analyzed the quality and quantity of volatiles from of three growth stage, as well as identified few compounds that attract or repel the *B. tabaci*. The results demonstrated that the distinct volatiles are emitted by pre-flowering, flowering, and fruiting plants with varying effects on *B. tabaci* preference. Three volatile compounds (linalool, (Z)-3-hexenyl acetate, and nonanal) analyzed in this study had trapping/repellent effects on *B. tabaci.* Therefore, these compounds can be adopted as potential attractants or repellents to control *B. tabaci*.

**Abstract:**

*Bemisia tabaci* Gennadius (Hemiptera: Aleyrodidae) is a worldwide pest that damages over 900 host plant species. The volatile organic compounds (volatiles) of contrasting plants, as well as their growth stage, influence this pest’s infestation behavior. The chemical contents of volatiles isolated from three plants (*Gossypium hirsutum*, *Abutilon theophrasti*, and *Ricinus communis*) during various growth phases (pre-flowering, fluorescence, and fruiting) were examined, as well as their influence on the behavior of adult *B. tabaci*. The olfactometer studies demonstrated that growth periods of the three plants affected the preference of *B. tabaci*. Volatiles of piemarker and cotton plants had dissimilar levels of attraction to adults during all stages. Volatile substances released by the castor at the stage of flowering had repellent effect on *B. tabaci*. In the plant versus plant combination, piemarker volatiles before and during anthesis were most preferred by adults, followed by cotton and then castor. A total of 24, 24, and 20 compounds were detected from volatiles of piemarker, cotton, and castor, respectively, and proportions among the compounds changed during different stages of plant development. The olfactory responses of *B. tabaci* to volatile compounds presented that linalool and high concentration of (Z)-3-hexenyl acetate had a strong trapping effect on this pest, while nonanal had a significant repellent effect at high concentration. This study indicates that distinct plants and their growth stage affect their attractiveness or repellency to *B.* *tabaci* adults, which are mediated by changing plant volatiles. These compounds obtained by analysis screening can be adopted as potential attractants or repellents to control Mediterranean (MED) *B. tabaci.*

## 1. Introduction

*Bemisia tabaci* Gennadius (Hemiptera: Aleyrodidae) is a worldwide pest that is known to damage more than 900 host plant species [1]. It was first observed in tobacco in Greece in 1889 [2,3]. *Bemisia tabaci* is a species complex with more than 40 cryptic species. Middle East-Asia Minor I (MEAM1, formerly known as the B biotypes) and Mediterranean, (MED formerly known as the Q biotypes) are the two most invasive members of *B. tabaci* worldwide space [4]. Although chemical pesticides are still the main method to control *B. tabaci*, their widespread application has brought negative impacts on the environment and potential threats to human health [5]. Therefore, many scientific researchers are currently focused on how to take advantage of semiochemicals from plants to control this insect pest.

The complex interactions between plants and insects rely upon the volatiles released by the plants, and insects’ ability to specifically sense them for food, shelter, or oviposition [6,7]. Insects have a highly sensitive and specific olfactory system for plant volatiles [8]. The preference of herbivorous insects for host plants has been attributed to the presence of one or more attractive components in the host plant volatiles or the absence of repellent components [9].

Piemarker, *Abutilon theophrasti*, is Malvaceae family plant, is generally more attractive to some pests (*Sylepta derogata* and *Bemisia tabaci)* than cotton [5,10]. Castor *Ricinus communis*, which contains ricin, is used as an insecticide material worldwide, and in recent years has been applied to the agricultural control of *B. tabaci* [11,12]. However, recent studies and our field research have revealed that plants only attract or repel insects during a specific growth stage [13,14]. Such plant growth specific attraction and repulsion could be a result of the fluctuation of volatiles at various growth stages. However, very studies have been focused of plant growth specific volatile patterns. The push–pull strategy is used as an integrated pest management tool to changes the distribution and density through behavioral control of pests or natural enemies. It was first used in Australia in 1987 to control *Helicoverpa* sp. In recent years, there have also been some explorations aimed at using push–pull strategy for the control of the pest *B. tabaci* [1]. However, previous studies did not account the fact that the distinct growth periods of plants directly affect the efficiency of pushing and pulling. Hence, our study aimed to refine the optimal developmental period of plants employed in ‘push’ and ‘pull’. Results from this study can be accustomed to adjust the planting period of pushing and pulling plants in practical production and effectively boost the efficiency of *B. tabaci* control. In addition, compounds screened from different stages of the plant can employed in traps or avoidance agents in the field. Simplifying the integrated control method of whiteflies while increasing control efficiency.

The aim of this study is to determine (i) whether distinct growth stages of piemarker, cotton, and castor will attract or repel *B. tabaci* differentially; in doing so this study also collected and analyzed (ii) the volatiles released by these plants at different growth stages; furthermore, this study also characterized (iii) compounds that could attract or repel insects.

## 2. Materials and Methods

### 2.1. Plant Growth

The cotton (var.SGK321) seeds were gathered from the Institute of Plant Protection, Chinese Academy of Agricultural Sciences. China’s Wang Zheng seedling sales center provided piemarker plant seeds, while Shouguang wentian seed industry Co., Ltd. (Shouguang, China) supplied castor plant seeds. They were grown in pots (size: 13 cm in diameter) in a 2:1 mixture of seedling substrate and soil. All plants were nurtured in a greenhouse and tested after five true leaves came out. They were maintained at 28 ± 1 °C with 65–75% RH and a 16:8 (L:D) at a light intensity of 1400–1725 lux, no pesticides were applied to the pants before or during the experiment.

### 2.2. Insect Rearing

The *B. tabaci* (cryptic species MED), were reared on cotton plant (var.SGK321) in mesh cages (60 cm × 60 cm × 100 cm). They were maintained at 28 ± 1 °C with 65–75% RH and a 16:8 h (L:D). The *B. tabaci* was regularly identified by the mt DNA Co I gene sequence. Primers specific to the MED cryptic species: F-Primer 5′-3′ (CTTGGTAACTCTTCTGTAGATGTGTGTT) and R-Primer 5′-3′ (CCTTCCCGCAGAAGAAATTTTGTTC) were used for Co I sequencing.

### 2.3. Olfactory Choice Test with Y-Tube Olfactometer

The behavioral response of *B. tabaci* to volatile compounds from three plants was evaluated by Y-tube olfactometer bioassays, following the equipment and procedure as previously described by Akol et al. [15] and Saad et al. [16] with slightly modifications. The Y olfactometer is made of a transparent glass tube consisted of 7 cm long base with 0.8 cm internal diameter, the angle between the two 8 cm arms was 60°, and the two arms were connected to different odor sources by Teflon tubes. The air inlet is connected to the QC-3 atmospheric sampler (0.2–3 L/min, Beijing Municipal Institute of Labour Protection, Beijing, China), and the air outlet is connected to the Y-Tube. The air flow is first filtered through activated carbon, then humidified with distilled water and finally reaches the odor source (bottle with plants). The flow rate was set at 0.5 L/min.

The experiment was conducted in a dark room to prevent adults from receiving visual cues from the plants. The Y-tube olfactory meter(Glassware customization factory, Zhengzhou, China) was positioned 0.5 m vertically above a 300 lux LED light. The adults 3–4 days after emergence were released within 1.0 cm base of the Y-tube after 4 h starvation and their responses were assessed. If it crossed one-third of any of the two arms of the Y-tube branch in 3 min and stayed for at least 30 s and did not return, it was considered a positive-responsive individual. Otherwise, they were considered non-responsive insects. After testing ten insects, Y-tube olfactometer arms were inverted. A total of 50 adults were tested at the time from 8:00 a.m. in the morning to 18:00 p.m. in the evening. During the test, the temperature of the darkroom was kept at 26 °C and the humidity was about 65%. The combinations of plants tested are shown in Table 1.

### 2.4. Volatile Compounds Collection and Analysis

The collection apparatus for volatile emitted by pre-flowering, fluorescence, and fruiting stages of the three plants mentioned above involved a dynamic headspace collection system [17]. For volatiles, analysis plants along with the complete root system were recovered from the posts, and after wrapping the root in the aluminum foil, plants were transferred individually to 10 L cylindrical glass chambers. Before capturing volatiles, air filtered with activated charcoal entered the chamber at a rate of 1.0 L/min with a vacuum pump for more than 30 min. Plants were acclimated in the experiment arena for 1 h before volatile collection. In the course of the volatile collection, activated charcoal purified air was pumped through Teflon tubing into the system at a flow rate of 1 L/min, and exiting air with volatiles passed through a glass tube filled with 200 mg, 60–80 mesh Tenax TA adsorption column [18]. The samples were collected cyclically over a period of 4 h, and four plants (replicates) per treatment were harvested.

The trapped volatiles were extracted from the adsorbent tube utilizing 400 μL (4 times, 100 μL each time) n-hexane (Sinopharm Chemical Reagent Co., Ltd., Shanghai, China). The 7.03 ng/mL n-octane (Aladdin Co., Ltd., Shanghai, China) was added to piemarker and cotton samples as an internal standard [19,20], and 5.87 ng/mL methyl salicylate (Aladdin Co., Ltd., Shanghai, China) was added to castor sample as an internal standard [21]. The samples were stored at −20 °C until analyzed through GC-coupled mass spectrometry analysis (GC-MS)

The collected volatile samples were then analyzed using a gas chromatograph–mass spectrometer (Trace ISQ, Thermofisher, Marietta, CA, USA) equipped with a DB-5 MS column (30 m × 0.25 mm × 0.25 µm). Helium (99.999%) was adopted as a carrier gas with a flow rate of 1.0 mL min^−1^ with constant mode. Heating procedure: the column temperature was maintained to 50 °C for 1 min, the oven temperature was increased from 50 to 150 °C at a rate of 5 °C min^−1^ and held for 2 min, and then from150 to 250 °C at a rate of 10 °C min^−1^ and held for 2 min.

### 2.5. The Olfactory Responses of B. tabaci to Volatile Compound

By means of principal component analysis (PCA), the standard of three compounds with the highest Variable Importance in Projection (VIP) value among distinct phases of piemarker, cotton, and castor were selected to test the olfactory response of the *B. tabaci*. (Linalool, CAS: 78-70-6, Purity 95%; (Z)-3-hexenyl acetate, CAS: 3681-71-8, Purity 98%; and nonanal, CAS: 124-19-6, Purity 96%. All purchased from Aladdin Co., Ltd., Shanghai, China). The three standards were diluted with n-hexane solution of 100, 10, and 1 (μL/mL), correspondingly. Diluted solution of the standard (20 microliters) was dropped on 1.5 cm × 2 cm qualitative filter paper and put into the bottle connected by one arm of the “Y” shaped tube. As a control, a quality filter paper soaked in 20 microliters of n-hexane was placed in the bottle on the opposite arm. Choice test with Y-Tube olfactometer was conducted using the procedure described above.

### 2.6. Data Analysis

IBM SPSS 25.0 was applied to conduct all statistical analyses. For the Y-tube olfactory test, the null hypothesis was that the pest would prove to have no preference (i.e., a 25:25 response) for each arm. Data produced from Y-tube olfactometer choice bioassays were analyzed by χ^2^ tests. Those who did not choose a host plant were excluded from the selection process. Besides, the compounds were identified by (i) comparing their retention times and mass spectra with authentic standards, (ii) comparing their mass spectra relative to those calculated for C8-C20 n-alkanes on DB-5 columns, and (iii) comparing their mass spectra for volatiles in the retention index and mass spectral library NIST 2014 (National Institute of Standards and Technology, Washington, DC, USA) database. Exploration of the GC data collected from a different stage of piemarker, cotton, and castor samples were preliminarily conducted by principal component analysis (PCA). Classification models using partial least square-discriminant analysis (PLS-DA) were generated and validated. Class modelling of three plants were finally performed by Soft Independent Model Class Analogy (SIMCA), respectively, to test the differences in the plant volatiles of different growth stage [22].

## 3. Results

### 3.1. Response of B. tabaci to Volatiles of Pre-Flowering Plants

**Figure 1:**
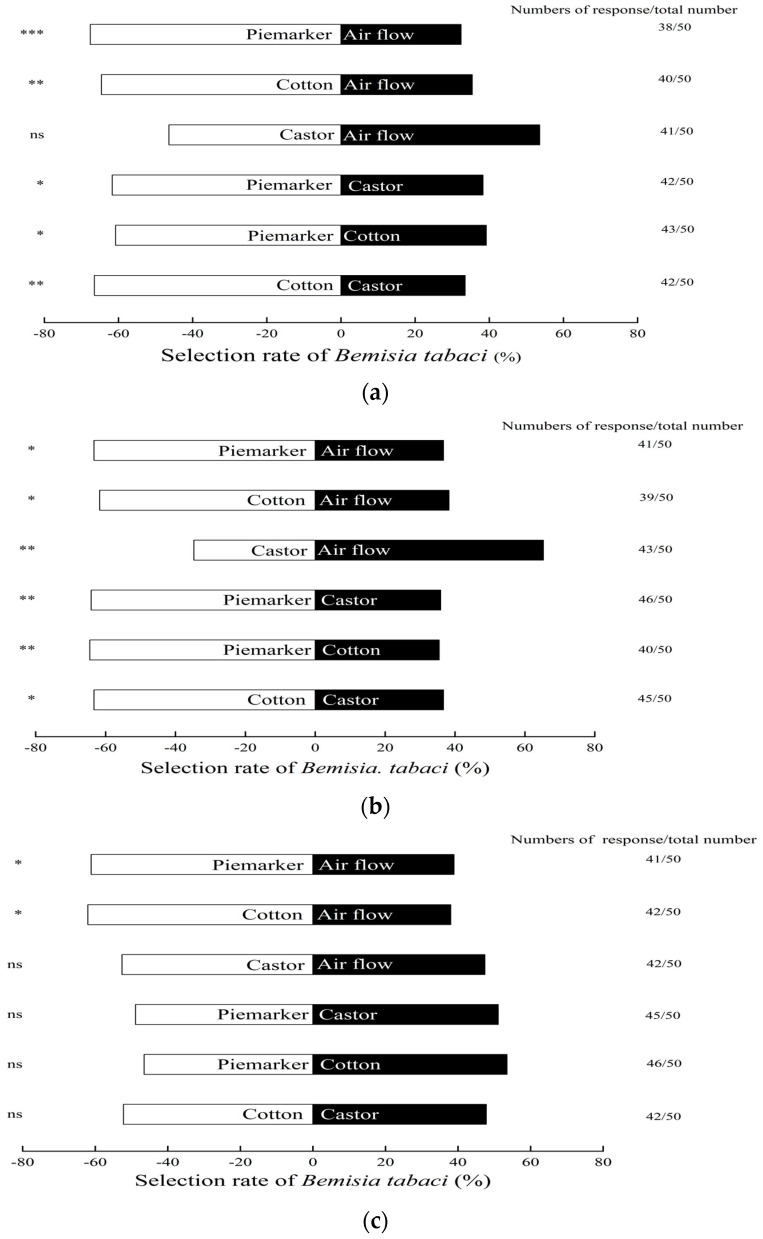
Attracting effects of different host plants on *Bemisia tabaci* at (**a**) pre-flowering, (**b**) flowering, and (**c**) fruiting stage. The data in the histogram represents the percentage of individuals who acted in each treatment. * Significant difference (*p* < 0.05), ** extraordinarily significant difference (*p* < 0.01), *** most significant difference (*p* < 0.001), and ns shows no significant difference (*p* > 0.05), *χ*^2^ test.

The statistical analysis indicated *B. tabaci* preferentially oriented towards volatiles from piemarker and the cotton at the pre-flowering stage compared to air. The selection rates of the adults on piemarker and cotton were 67.6% (*χ*^2^ = 11.7, *p* < 0.001) and 64.6% (*χ*^2^ = 7.96, *p* < 0.01) respectively. However, there was no obvious reaction between caster at the pre-flowering stage (46.4% *χ*^2^ = 0.38, *p* > 0.05) and air flow (Figure 1a).

The *B. tabaci* responded preferentially to volatiles emitted by piemarker (61.7%, *χ*^2^ = 5.02, *p* < 0.05), when compared to castor (38.3%). They also responded favorably to volatiles from piemarker space (60.8%, *χ*^2^ = 4.26, *p* < 0.05), in comparison with cotton (39.2%). The distinction between cotton and castor was exceptionally significant, in which 66.5% of *B. tabaci* chose cotton, and only 33.5% of them selected castor (*χ*^2^ = 10.24, *p* < 0.01) (Figure 1a).

### 3.2. Response of B. tabaci to Volatiles of Florescence Plants

During flowering-stage, the attraction of piemarker and cotton to *B. tabaci* was significantly higher than that of air flow, but castor appeared to be significantly more repellent to *B. tabaci*. Compared to air, the selection rate of *B. tabaci* on piemarker was 63.3% (*χ*^2^ = 6.56, *p* < 0.05), on cotton it was 61.7% (*χ*^2^ = 5, *p* < 0.05), and on castor it was 34.7% (*χ*^2^ = 8.76, *p* < 0.01) (Figure 1b).

In the comparison of the three plants in florescence stage, the *B. tabaci* always favored piemarker more, while it maintained the weakest preference for castor. Between the combinations of piemarker and castor, the selection rate of *B. tabaci* for the former and the latter was 64.1% and 35.9% (*χ*^2^ = 7.36, *p* < 0.01), respectively. In the piemarker versus cotton, 64.5% of the adults chose piemarker and 35.5% chose cotton (*χ*^2^ = 7.84, *p* < 0.01). There were also significant differences between cotton and castor. Among them, 63.3% of *B. tabaci* responded favorably to volatiles from cotton, while only 36.7% to castor (*χ*^2^ = 6.56, *p* < 0.05) (Figure 1b).

### 3.3. Response of B. tabaci to Volatiles of Fruiting Plants

The *B. tabaci* responded preferentially to volatiles from piemarker and cotton when compared to air, respectively, but there was no significant difference between castor and air (Figure 1c). Compared to air, the selection rate of *B. tabaci* was 61.1% for piemarker (*χ*^2^ = 4.5, *p* < 0.05), 62.0% for cotton (*χ*^2^ = 5.3, *p* < 0.05), and 52.6% for castor (*χ*^2^ = 4.5, *p* > 0.05), respectively.

The *B. tabaci* adults had no significant preference to the volatiles between piemarker (48.9%) and castor plants (51.1%, *χ*^2^ = 0.02, *p* > 0.05), and between piemarker (46.5%) and cotton plants (53.5%, *χ*^2^ = 0.36, *p* > 0.05), as well as between cotton (52.2%) and castor (47.8%, *χ*^2^ = 0.12, *p* > 0.05) (Figure 1c).

### 3.4. Qualitative Analysis of Three Plant Volatiles in Different Periods

In total, 24 volatiles were identified from the piemarker plants, with 22 detected before flowering, 23 at flowering, and 19 at fruiting. Similarly, 24 compounds were identified from three growth stage of cotton. The pre-flowering, florescence, and fruiting stages of cotton emitted 20, 23, and 20 compounds, respectively. A total of 20 compounds were detected in castor during the three stages, including 15 at pre-flowering, 19 at flowering, and 16 at the fruiting stage (Supplementary Figure S1).

### 3.5. PLS-DA Analysis of Different Periods Volatiles of Three Plants

**Figure 2:**
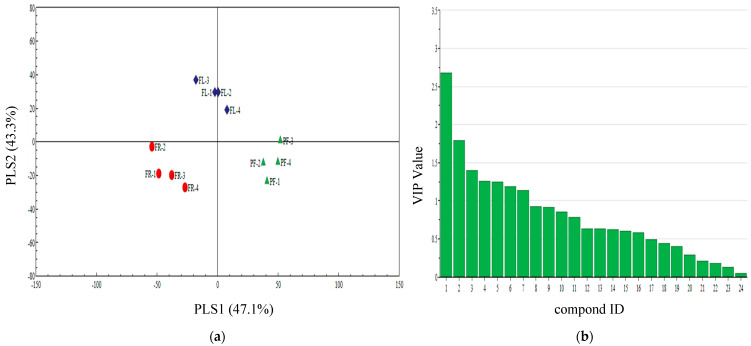
(**a**) Partial least squares discriminant analysis (PLS-DA) of pre-flowering (PF), florescence (FL), and fruiting (FR) of piemarker plant volatile compounds. The score plot display the grouping pattern according to the first two components and the ellipse defines the Hotelling’s T^2^ confidence interval (95%) for the observations. (**b**) VIP value of piemarker plant volatile compounds. Compounds ID: (1) linalool, (2) nonane, (3) ethyl octanoate, (4) ethyl nonanoate, (5) butyl acrylate, (6) 1,3,7-ocimene, (7) 3-hexadecanol, (8) ethyl decanoate, (9) decane, (10) octanal, (11) 2-ethyl hexanol, (12) 2-butyl-1-octanol, (13) naphthalene, (14) di-n-butyl ether, (15) nonanal, (16) Decanal, (17) 6-methyl-5-hepten-2-one, (18) α-caryophyllene, (19) 1-hexanol, (20) 2-ethylhexyl acrylate, (21) ethylbenzene, (22) methyl benzoate, (23) dodecyl aldehyde, and (24) 3-hexadecanol.

The partial least squares-discriminant analysis (PLS-DA) showed that the volatiles contents were clearly separated among the pre-flowering, florescence, and fruiting stages of piemarker. The first two significant PLS components explained 43.3% and 47.1% of the total variance, respectively (Figure 2a). In this model, the following seven volatile compounds including linalool, nonane, ethyl octanoate, ethyl nonanoate, butyl acrylate, 1,3,7-ocimene, and 3-hexadecanol with VIP values ≥1.0 contributed most to the separation among different periods volatiles of piemarker (Figure 2b).

**Figure 3:**
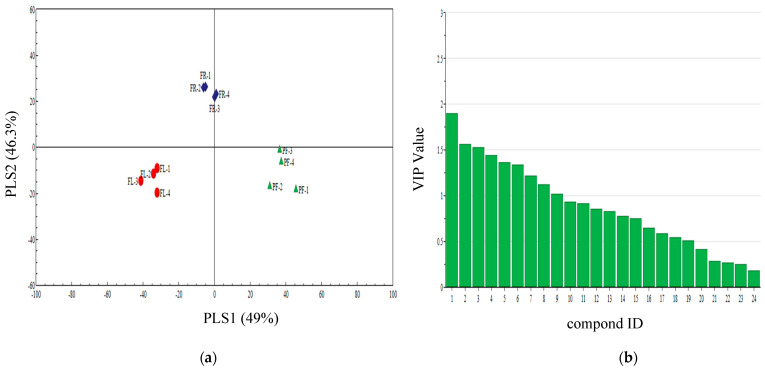
(**a**) Partial least squares discriminant analysis (PLS-DA) of pre-flowering (PF), florescence (FL), and fruiting (FR) of cotton plant volatile compounds. The score plot display the grouping pattern according to the first two components and the ellipse defines the Hotelling’s T^2^ confidence interval (95%) for the observations. (**b**) VIP value of cotton plant volatile compounds. Compounds ID: (1) (Z)-3-hexenyl acetate, (2) 2-hexenal, (3) ethyl octanoate, (4) naphthalene, (5) ethyl nonanoate, (6) nonane, (7) 1,3-xylene, (8) dodecyl aldehyde, (9) octanal, (10) α-caryophyllene, (11) linalool, (12) ethylbenzene, (13) DMNT = (3E)-4,8-dimethyl-1,3,7-nonatriene, (14) 6-methyl-5-hepten-2-one, (15) α-pinene, (16) 3,7-dimethyl-1,3,6-octatriene, (17) caryophyllene, (18) decanal, (19) nonanal, (20) 2-ethylhexyl acrylate, (21) methyl benzoate, (22) benzaldehyde, (23) decane, (24) ethyl decanoate.

The PLS-DA also showed a clear separation among pre-flowering, florescence, and fruiting of cotton. The first two significant PLS components explained 46.3% and 49% of the total variance, respectively (Figure 3a). The first component and the second component showed a clear separation among three volatiles of different periods of cotton. In this model, the following nine volatile compounds: (Z)-3-hexenyl acetate, 2-hexenal, ethyl octanoate, naphthalene, ethyl nonanoate, nonane, 1,3-xylene, dodecyl aldehyde, and octanal with VIP values ≥ 1.0 contributed most to the separation among different periods volatiles of cotton (Figure 3b)

**Figure 4:**
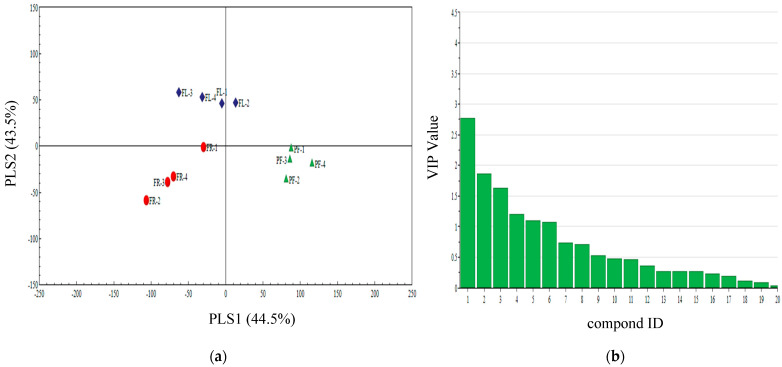
(**a**) Partial least squares discriminant analysis (PLS-DA) of pre-flowering (PF), florescence (FL), and fruiting (FR) of castor plant volatile compounds. The score plot display the grouping pattern according to the first two components and the ellipse defines the Hotelling’s T2 confidence interval (95%) for the observations. (**b**) VIP value of castor plant volatile compounds. Compounds ID: (1) nonanal, (2) ethyl decanote, (3) 1-octanol,2-butyl-, (4) butyl acrylate, (5) 3-hexadecanol, (6) naphthalene, (7) ethylbenzene, (8) 3,7-dimethyl-1,3,6-octatriene, (9) decanal, (10) butyl acetate, (11) dodecyl aldehyde, (12) 1,3-xylene, (13) d-longifolene, (14) 2-ethyl hexanol, (15) methyl benzoate, (16) (-)-thujopsene, (17) 6-methyl-5-hepten-2-one, (18) di-n-butyl ether, (19) Decane, (20) α-pinene.

The PLS-DA also showed a clear separation among pre-flowering, florescence, and fruiting of castor. The first two significant PLS components explained 43. 5% and 44.5% of the total variance (Figure 4a). In this model, the following six volatile compounds nonanal, ethyl decanote, 2-butyl-1-octanol, butyl acrylate, 3-hexadecanol, and naphthalene with VIP values ≥1.0 contributed most to the separation among different periods volatiles of castor (Figure 4b).

### 3.6. The Olfactory Responses of B. tabaci to Standard Samples of Volatile Compound

**Figure 5:**
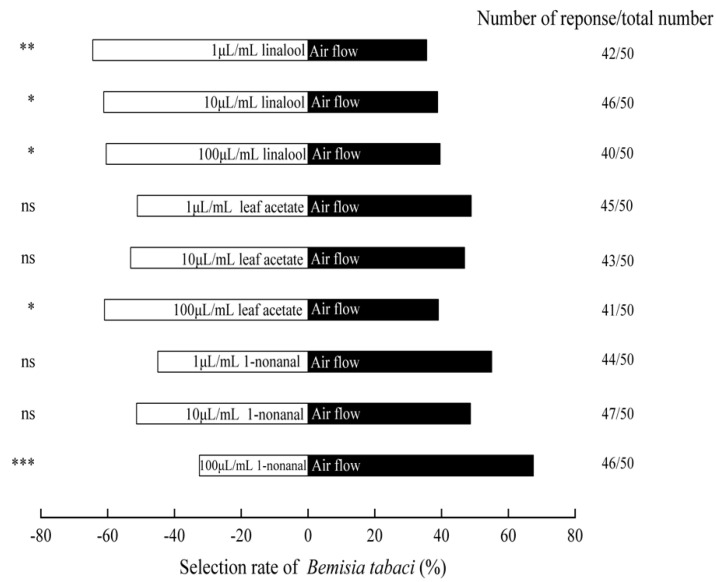
The data in the histogram represent the percentage of individuals who acted in each treatment. * Significant difference (*p* < 0.05), ** extraordinarily significant difference (*p* < 0.01), *** most significant difference (*p* < 0.001), and ns shows no significant difference (*p* > 0.05), *χ*^2^ test.

The Figure 5 showed that among the three compounds, the *B. tabaci* had clear preference to linalool. The linalool concentrations at 1, 10, and 100 μL/mL attracted 64.4% (*χ*^2^ = 7.72, *p* < 0.01), 61.2% (*χ*^2^ = 4.56, *p* < 0.05), and 60.4% (*χ*^2^ = 3.92, *p* < 0.05) adults, respectively. In addition, (Z)-3-hexenyl acetate was also significantly attractive to this pest at 100 μL/mL, and the selection rate was 60.9% (*χ*^2^ = 4.32, *p* < 0.05). However, there was no significant difference between air and 1 and 10 μL/mL (Z)-3-hexenyl acetate (*χ*^2^ = 0.02, *p* > 0.05; *χ*^2^ = 0.28, *p* > 0.05). At a concentration of 100 μL/mL, nonanal showed the obvious repellant effects against *B. tabaci*, and the selection rate was only 32.5% (*χ*^2^ = 11.56, *p* < 0.001). However, there was no significantly preference of adults to medium and low concentrations of nonanal (*χ*^2^ = 1.87, *p* > 0.05; *χ*^2^ = 0.02, *p* > 0.05).

## 4. Discussion

In this study, we investigated the response of *B. tabaci* to chemical cues released by different plants at their divergent growth stages. According to the results, the piemarker plants attracted *B. tabaci* adults, while the castor repelled them. However, attraction/avoidance only occur during a certain growth period of plants, which was closely related to the volatiles released by plants.

Besides, our study indicates that the physiological stage of the host plant influences the host-foraging behavior of the whitefly. The “Y” olfactometer test revealed that piemarker is more attractive to *B. tabaci* in the pre-flowering stage. At the pre-flowering and flowering stages of the plant versus plant treatment, the majority of adults chose piemarker, followed by cotton, and then castor. However, the preference for *B*. *tabaci* in the three plants was not significantly different during the fruiting stage, which suggests that the piemarker, as an attractor of pests, was more attractive at the pre-flowering and flowering stages. Mohammed et al. studied the influences of the volatiles from different parts of brinjal plants on the behavior of adult *Leucinodes orbonalis* and found that adults responded differently to the volatiles extracted from fruits, leaves, shoots, and flowers [23]. It is worth noting that we detected that the castor had a repellent effect on *B. tabaci* at the flowering stage. As presented in the preference test viz the castor vs. airflow treatment, only 35.9% of adults chose castor at the flowering stage, while there was no preference of *B. tabaci* detected at the pre-flowering stage and fruiting stage. Moreover, Luo et al. investigated the population of *B. tabaci* in the castor trap belt, its neighboring cotton fields, and castor-free cotton fields, and found that there was no significant difference in the number of *B. tabaci* in the three fields between the months of June and July [12]. However, during the months of August and September, the number of whiteflies on castor and cotton adjacent to castor was 1205 and 1580 per 100 plants, respectively, significantly lower than that of control cotton (4697 per 100 plants). Therefore, low abundance of *B. tabaci* in cotton planted with castor might be the result of repellent effects of castor. Data from this study revealed that, in the castor–cotton–piemarker push–pull strategy, the effective period for ‘pushing’ plants in castor is the flowering period, whereas effective growth stages for ‘pulling’ plants in piemarker are the pre-flowering and flowering periods. Therefore, planting time of castor and piemarker can be adjusted on the basis of the sowing time of cotton to achieve the best prevention effect against *B. tabaci*.

The host-seeking behavior of whiteflies may be caused by the fluctuating composition and concentration of volatiles at various stages of plant growth. The volatiles play a key role in the process of host recognition, which is an important clue for insects to identify and locate food and natural enemies [24]. In this study, over 30 different compounds were identified, including alcohols, aldehydes, esters, and terpenes (24 in piemarker, 24 in cotton, and 20 in castor). This is almost in line with previous reports [19,25,26]. Likewise, the proportion of some volatile compounds in the three plants changed significantly at divergent growth stages, which may be the reason for the differences in preference for *B. tabaci* at distinct growth stages.

In the current study, the olfactory responses of *B. tabaci* to volatile compounds, such as linalool, (Z)-3-hexenyl acetate, and nonanal, were determined. It was discovered that linalool and a high concentration of linalool acetate had powerful trapping effects on *B. tabaci* MED, while nonanal had significant repellent effects at high concentration. Similarly, Li reported that linalool at concentrations of 1, 0.01, and 0.0001 μL/μL had a significant effects on the attraction of *B. tabaci* MED [27]. Besides, linalool also has a seductive effect on various pests, such as *Holotrichia online*, *Rhopalosiphum padi*, *Frankliniella intonsa,* etc. [28,29,30], which can have a multiplier effect on the attraction of pests. Stevens et al. reported that the release of (Z)-3-hexenyl acetate in citrus orchards had an attractive effect on *Parastethorus nigripes* and *Stethorus vagans* [31]. Furthermore, (Z)-3- hexenyl acetate can be used as a sex pheromone potentiator to attract *Grapholita molesta* males to pheromone traps [32,33]. Previous studies have reported that nonanal has a repellant effect on certain insects and can be employed as repellent in a push–pull strategy [34,35]. Similar to our report, previous studies found that the concentration of the compounds is also a significant factor affecting host selection by insects [35]. In summary, data indicate that linalool, (Z)-3-hexenyl acetate, and nonanal can be utilized as potential attractants or repellents to control *B. tabaci*. However, our trials are conducted in the laboratory and further field trials of individual and combined compounds are warranted to confirm the effects of linalool, f(Z)-3-hexenyl acetate, and nonanal on *B. tabaci* MED.

## 5. Conclusions

This study indicates that depending upon the growth stage, piemarker and castor can be used in B. tabaci management programs in the cotton ecosystem. However, future field studies are warranted on the effects of these crops on the overall pest management program in cotton. In addition, this study also identified three volatile compounds (linalool, (Z)-3-hexenyl acetate, and nonanal) that could be used as potential attractants or repellents.

## Figures and Tables

**Table 1:** Test grouping.

Treatment		Treatment	Treatment		Treatment
pre-flowering piemarker	VS	air flow	pre-flowering piemarker	VS	pre-flowering cotton
pre-flowering cotton	VS	air flow	pre-flowering piemarker	VS	pre-flowering castor
pre-flowering castor	VS	air flow	pre-flowering cotton	VS	pre-flowering castor
florescence piemarker	VS	air flow	florescence piemarker	VS	florescence cotton
florescence cotton	VS	air flow	florescence piemarker	VS	florescence castor
florescence castor	VS	air flow	florescence cotton	VS	florescence castor
fruiting piemarker	VS	air flow	fruiting piemarker	VS	fruiting cotton
fruiting cotton	VS	air flow	fruiting piemarker	VS	fruiting castor
fruiting castor	VS	air flow	fruiting cotton	VS	fruiting castor

## Data Availability

All data generated or analysed during this study are included in this published article and its Supplementary information files.

## Supplementary Materials

The following supporting information can be downloaded at: https://www.mdpi.com/article/10.3390/insects13080703/s1, Figure S1: Qualitative Analysis of Three Plant Volatiles in Different Periods.
