# Supplementary figures and images for: Behavioral Responses of Bemisia tabaci Mediterranean Cryptic Species to Three Host Plants and Their Volatiles
Source: Insects. 2022 Aug 5;13(8):703. doi: 10.3390/insects13080703 (PMC9409411; doi:10.3390/insects13080703)

Figure S1. Qualitative Analysis of Three Plant Volatiles in Different Periods

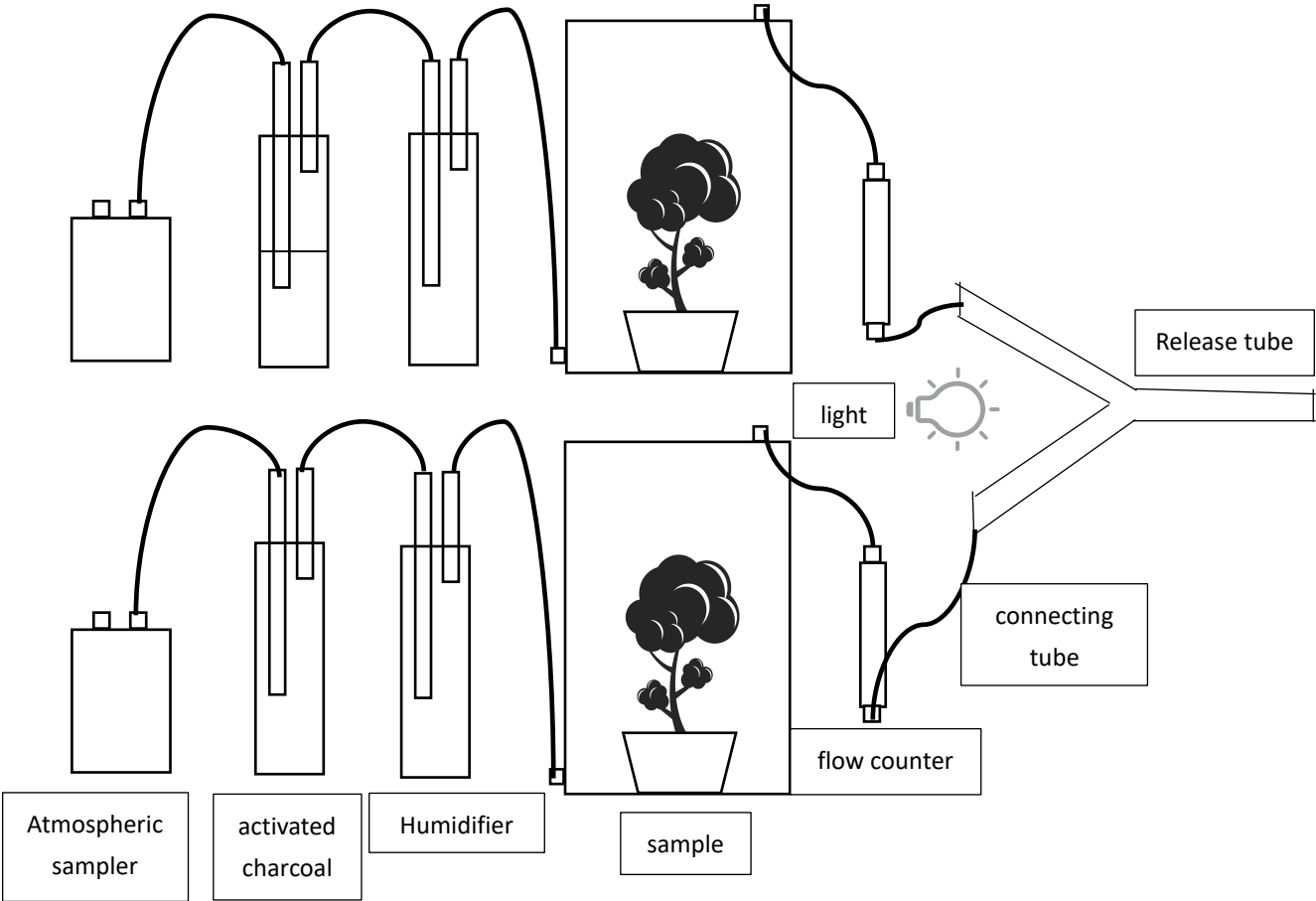

Supplement: Supplementary file 1 [file insects-13-00703-s001.zip › insects-1805406-supplementary.pdf]
